# Verifying the validity and reliability of the Japanese version of the Face, Legs, Activity, Cry, Consolability (FLACC) Behavioral Scale

**DOI:** 10.1371/journal.pone.0194094

**Published:** 2018-03-13

**Authors:** Yujiro Matsuishi, Haruhiko Hoshino, Nobutake Shimojo, Yuki Enomoto, Takahiro Kido, Tetsuya Hoshino, Masahiko Sumitani, Yoshiaki Inoue

**Affiliations:** 1 Department of Emergency and Critical Care Medicine, Faculty of Medicine, University of Tsukuba, Tsukuba, Ibaraki, Japan; 2 Pediatric Intensive Care Unit, University of Tsukuba Hospital, Tsukuba, Ibaraki, Japan; 3 University of Tsukuba Hospital, Department of Pediatrics, Tsukuba, Ibaraki, Japan; 4 Department of Anesthesiology and Pain Relief Center, University of Tokyo Hospital, Tokyo, Japan; Tokyo Metropolitan Institute of Medical Science, JAPAN

## Abstract

**Background:**

Pediatric patients, especially in the preverbal stage, cannot self-report intensity of pain therefore several validated observational tools, including the Face, Legs, Activity, Cry, Consolability (FLACC) Behavioral Scale, have been used as a benchmark to evaluate pediatric pain. Unfortunately, this scale is currently unavailable in Japanese, precluding its widespread use in Japanese hospitals.

**Objectives:**

To translate and verify the validity and reliability of the Japanese version of the FLACC Behavioral Scale.

**Method:**

Back-translation was first conducted by eight medical researchers, then an available sample of patients at the University of Tsukuba Pediatric Intensive Care Unit (from May 2017 to August 2017) was enrolled in a clinical study. Two researchers evaluated the validity of the translated FLACC Behavioral Scale by weighted kappa coefficient and intraclass correlation coefficients (ICC). Observational pain was simultaneously measured by the visual analog scale (VAS obs) and reliability was evaluated by correlation analysis.

**Result:**

The original author approved the translation. For the clinical study, a total of 121 observations were obtained from 24 pediatric patients. Agreement between observers was highly correlated for each of the FLACC categories (Face: κ = 0.85, Leg: κ = 0.74, Activity: κ = 0.89, Cry: κ = 0.93, Consolability: κ = 0.93) as well as the total score (Total: κ = 0.95,). Correlation analysis demonstrated a good criterion validation between the FLACC scale and the VAS obs. (r = 0.96)

**Conclusion:**

Our Japanese version of the FLACC Behavioral Scale shows high validity and reliability.

## Introduction

Relief of pain is a basic human right regardless of expressive ability and, in a concerning trend, several studies have reported that patients in the pediatric intensive care unit (PICU) [1,2]require more invasive procedures compared to the general ward. Additionally, painful procedures such as heel sticks and venous arterial punctures are frequently performed in PICU which would logically indicate higher pain levels in these settings [2]. However, pediatric nurses are often challenged to identify pain at the preverbal development stage and efforts to do so are further complicated in critically ill patients undergoing sedation and mechanical ventilation. To solve this situation, several validated observational tools, including the Face, Legs, Activity, Cry, Consolability (FLACC) Behavioral Scale [3], have been developed for pediatric patients in intensive care settings. The FLACC Behavioral Scale has the advantages of both wide recognition and distribution (it is available in several languages) and previous studies have reported high reliability and validity in assessing acute pain for pediatric patients [3,4]. However, to this point in time, reliable assessment tools for detecting pediatric pain, such as the FLACC Behavioral Scale, have been unavailable in Japanese hospitals due to language barriers. Thus, the aims of the present study are to translate the FLACC Behavioral Scale using the back-translation method and to analyze the reliability and validity of this new Japanese version.

## Methods

### Translation

**Fig 1:**
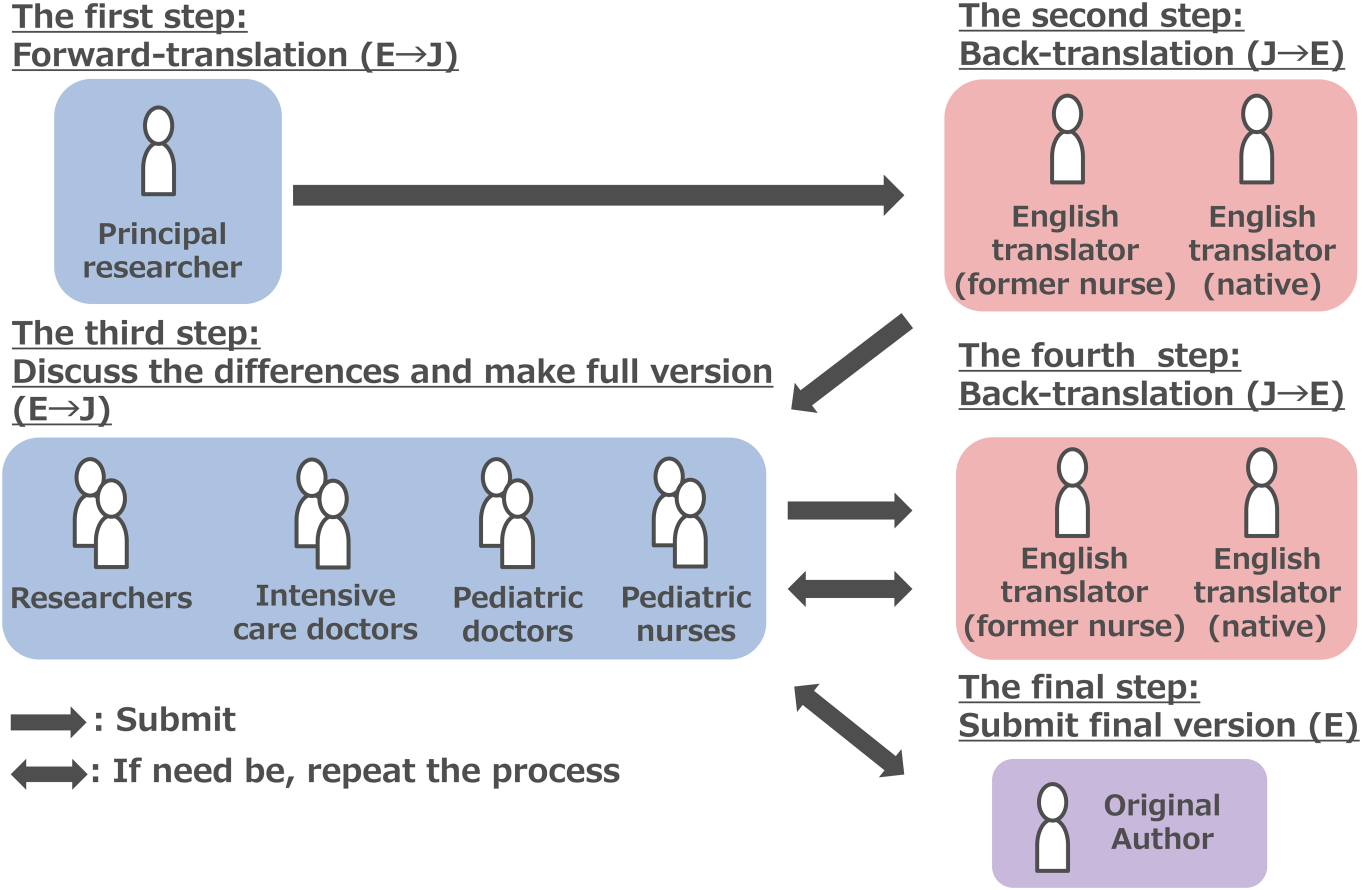
Back translation method. Flow of the back translation method used to translate the Face, Legs, Activity, Cry, Consolability (FLACC) Behavioral Scale.

Prior to the beginning of the study, written permission to translate the FLACC Behavioral Scale was obtained from the developer (Ms. Sandra Merkel) and we received an Academic/Non-Profit license from the University of Michigan. Translation was conducted using the back-translation method. This method is a widely accepted method that maintains the overall literature and meaning between the original and translated versions. The translation process of the FLACC Behavioral Scale was as follows (Fig 1).

In the first step, the principal researcher created a tentative English to Japanese version. Next, we submitted this tentative version to a second set of translators that consisted of both a Japanese who had been a nurse in the U.S. and a native speaker of American English. In the third step, eight medical workers (including two clinical researchers, two intensive medical doctors, two pediatric doctors and two nurses working at PICU) discussed the differences observed in all individual translations, back translated the document from English to Japanese, and then resubmitted this to the translators described above. For consistency in translation as well as reduction in variability between multi-disciplinary medical staff, eight local medical workers carefully checked any possible differences between the original and back-translated versions. Every effort was made to carefully execute all the steps in order to avoid the loss of the original content due to cultural differences. After completion, the final document was then checked and approved by the original author (Ms. Sandra Merkel). Technical details of these process was shown in our previous reports [5].

The second and third translation steps previously described above were repeated once. Although minor changes between the tentative and completed versions were needed to address nuances in Japanese meaning, there were no major changes. The completed version was checked and confirmed by the original author and sited on website [6].

### Validation and reliability study

We performed a validation and reliability study using our newly-established Japanese version of the FLACC Behavioral Scale. We enrolled a number of patients from the PICU at the University of Tsukuba Hospital from May to August, 2017 on every Wednesday, and we exclude patient using muscle relaxants. We recorded baseline characteristics, including age, sex, diagnosis for PICU admission, ventilation status, withdrawal syndrome as assessed by The Withdrawal Assessment Tool—Version 1 (WAT-1) [7], delirium as assessed by the Cornell Assessment of Pediatric Delirium (CAPD) [8] and severity calculated by Pediatric Index of Mortality 2 (PIM2) [9]. Additional evaluation of the FLACC Behavioral Scale was done by two researchers who objectively and simultaneously measured pain by the observational visual analog scale (VAS obs) for each patient. VAS obs is the method which observers estimate subject symptoms by observation. Using VAS obs for neonate and child is previously reported [10,11] and Correration between FLACC Behavioral Scale and VAS obs is measured by correration analysis. Acoording to Guilford’s Rule of Thumb [12], we consider correlation coefficients of less than 0.20 as "slight almost negligible relationships", 0.20 to 0.40 as "low correlation;" 0.40 to 0.70 as "moderate correlation;" 0.70 to.90 as "high correlation" and greater than 0.90 as "very high correlation". Main researcher was blind to the score of the other and VAS obs was evaluated before the FLACC Behavioral Scale to remove any bias.

### Sample size

Adequate sample size and variability change depending on the cohort. Thus, we calculated our needed sample size based on reliability as previously published [13]. Based on this previous study [13], agreement between observers is taken as an estimate of strong correlation (r = 0.7). We determined that a sample size of 17 patients would be required for a significance level (α) of 0.05 and test power (1-β) of 0.90 [14].

### Statistics

Agreement between observers for each of the five FLACC categories was evaluated by weighed Cohen’s kappa coefficient which is commonly used for summarizing the cross-classification of ordinal variables with identical categories [15]. It allows the use of weights to describe the closeness of agreement between categories. We additionally examined inter-rater agreement (concordance) by the widely-used intraclass correlation coefficient (ICC) [16] that contains 10 model groups that can be chosen based on purpose [17]. For this study, we selected the two-way random-effects model (absolute agreement with multiple raters/measurements (2, k)) [18] to generalize our reliability results.

To assess the validity criterion, agreement between VAS obs and the FLACC Behavioral Scale was evaluated by correlation analysis. All statistical analyses were performed using SPSS version 24 (SPSS, Inc., Chicago, IL). Values under 0.05 were considered statistically significant.

### Ethics

This study was approved by the Institutional Review Board (IRB) of the University of Tsukuba Hospital and written informed consent was obtained from patients or legally designated representatives (such as family) prior to study.

## Result

### Characteristics

From May to August, 2017, total of 121 observations were obtained from 24 pediatric patients. Table 1 presents baseline patient study characteristics.

**Table 1:** Baseline characteristics of study patients.

variable	N = 24
Age (m) ± SD	40 ± 50
Male n(%)	13 (54)
Diagnosis	
Cardiac surgical n (%)	13 (54)
Abdominal Surgical n (%)	6 (25)
Neuro surgical n (%)	3 (12)
Thoracic surgery n (%)	1 (4)
Medical n (%)	1 (4)
PIM2	3.2 ± 5.2
Mechanical ventilation ^a^	12 (50)
Delirium ^b^	8 (30)
Withdrawal syndrome ^c^	0 (0)

^a^: At least one time treated with Mechanical ventilation when the observation.

^b^: At least one time experience delirium detected by CAPD when the observation.

^c^: At least one time experience Withdrawal syndrome detected by WAT-1 when the observation.

SD = standard deviation, PIM2 = Pediatric index of mortality 2

The median age at enrollment was 38 months (± 47), 45% of the patients were male and 50% of the total pool of patients received at least one day of mechanical ventilation. The PIM2 average was 1.6 (± 5.4) and the prevalence of delirium was 30%. No withdrawal syndrome was noted in any patient. The primary medical diagnosis for PICU admission was cardiac surgery (45%).

### Reliability

Agreement between observers was highly correlated for each of the FLACC categories (Face: κ = 0.85, 95%CI [0.73–0.96], Leg: κ = 0.74, 95%CI [0.55–0.94], Activity: κ = 0.89, 95%CI [0.73–1.0], Cry: κ = 0.93, 95%CI [0.8–1.0], Consolability: κ = 0.93, 95%CI [0.8–1.0]) as well as total score (Total: κ = 0.95, 95%CI [0.91–0.98]). The categories of Cry and Consolability show the highest agreement between observers. The reliability of the FLACC Behavioral Scale is slightly higher in patients who did not receive mechanical ventilation versus those who did (Non-Mechanical Ventilation group: κ = 0.93, 95%CI [0.86–1.0] vs. Mechanical Ventilation group: κ = 0.91, 95%CI [0.83–0.99]). Inter-rater agreement, as evaluated by ICC (2, k) calculations, returned a similar result to Cohen’s weighted Kappa coefficient. (Table 2)

**Table 2:** Reliability on Japanese version of FLACC Behavioral Scale.

variable	Statistics	Total number of times N = 121	With Mechanical ventilation N = 70	Without Mechanical ventilation N = 51
Face	weighted kappa ^a^	0.85 [0.73–0.96]	0.84 [0.68–1.0]	0.85 [0.7–1.0]
	ICC ^b^	0.92 [0.88–0.94]	0.91 [0.87–0.95]	0.92 [0.87–0.95]
Legs	weighted kappa ^a^	0.74 [0.55–0.94]	0.75 [0.58–0.92]	0.73 [0.33–1.0]
	ICC ^b^	0.85 [0.79–0.89]	0.86 [0.77–0.91]	0.84 [0.73–0.91]
Activity	weighted kappa ^a^	0.89 [0.73–1.0]	0.88 [0.64–1.0]	0.9 [0.68–1.0]
	ICC ^b^	0.94 [0.92–0.96]	0.93 [0.9–0.96]	0.95 [0.91–0.97]
Cry	weighted kappa ^a^	0.93 [0.8–1.0]	1 [1.0–1.0]	0.65 [0.01–1.0]
	ICC ^b^	0.96 [0.95–0.97]	1 [1.0–1.0]	0.79 [0.64–0.88]
Consolability	weighted kappa ^a^	0.93 [0.8–1.0]	0.9 [0.77–1.0]	1 [1.0–1.0]
	ICC ^b^	0.96 [0.95–0.97]	0.94 [0.91–0.96]	1 [1.0–1.0]
Total	weighted kappa ^a^	0.95 [0.91–0.98]	0.91 [0.83–0.99]	0.93 [0.86–1.0]
	ICC ^b^	0.97 [0.96–0.98]	0.97 [0.96–0.98]	0.97 [0.96–0.98]

^a^: Data are kappa coefficient [95% confidence interval]

^b^: Data are Intra class correlation coefficient [95% confidence interval]

### Criterion validity

**Fig 2:**
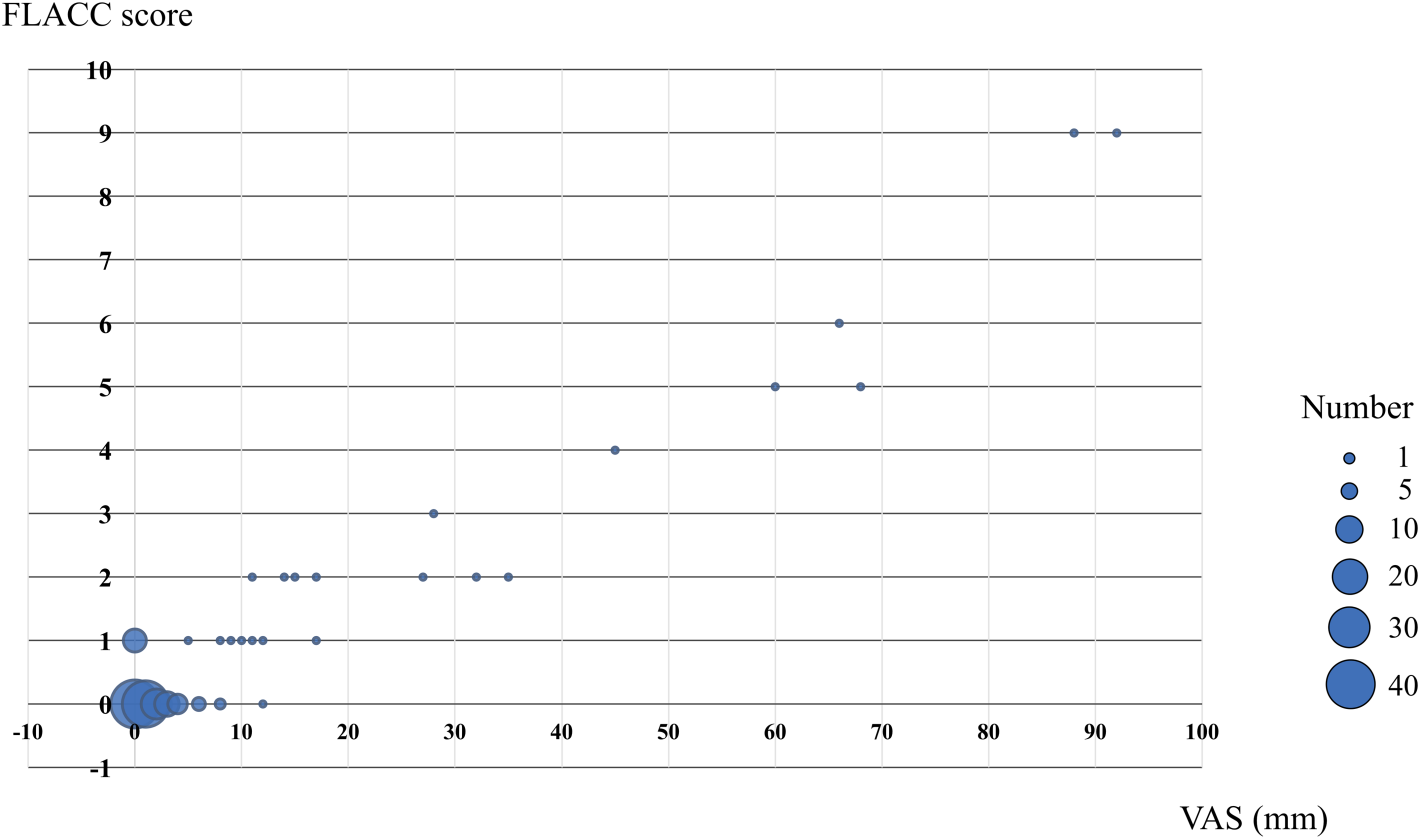
Criterion validity on Japanese version of FLACC Behavioral Scale. Correlation analysis between observational visual analog scale (VAS obs) and FLACC Behavioral Scale. FLACC Behavioral Scale score significantly correlated with VAS obs. (r = 0.96).

The FLACC Behavioral Scale score was very highly correlation with VAS obs (*r* = 0.96). (Fig 2).

Both of mechanically and non-mechanically ventilated patients were very highly correlation (Non-Mechanical Ventilation group: r = 0.96, Mechanical Ventilation group: *r* = 0.95).

## Discussion

The present study is the first to translate the FLACC Behavioral Scale from English to Japanese by using the back-translation method. As a previous study mentioned that direct translation does not guarantee sufficient equivalency [19], we therefore used the back-translation method and included a multi-disciplinary committee to remedy content variance. Of particular concern were medical terms and delicate nuances that might be hard to understand for laymen so we chose a Japanese nurse with certification and work experience in the U.S as well as a native speaker of American English. Additionally, we performed a criterion validation and reliability study for the completed translation. As language barriers often prevent useful medical evaluation standards from being propagated internationally, we hope that our present method could be applied to other medical translation efforts. In the original study, the FLACC Behavioral scale showed a high correlation between observers (*r* = 0.92), however diverse studies have shown a wide-ranging moderate to high reliability [20–22]. In this report, we show that our Japanese version has both high criterion validation and reliability in assessing pain for the patients in PICU. A previous study showed that the Cry category poorly correlated with other categories, most likely because of intubation [13]. Our results show high reliability (κ = 1.0, ICC = 1) in mechanically ventilated patients and relatively low reliability in non-mechanically ventilated patients (κ = 0.65, ICC = 0.79). This might be attributed to translation errors or cohort differences. As for translation, there are no cultural differences in the concept or language of crying between English and Japanese, so this could be ruled out. However, the fact that the primary diagnosis category of participants was cardiac surgery (45%) leads to the assumption that patients in need of mechanical ventilation might have a more severe condition that requires sedation. Thus, they are not vigorous enough to cry and are therefore more difficult to accurately assess in comparison with non-mechanically ventilated patients.

Correlation analysis demonstrated a solid criterion validation between the FLACC scale and the VAS obs (*r* = 0.92). In the previous studies, the FLACC Behavioral Scale was compared with other observable behavioral pain scales such as the Children’s Hospital of Eastern Ontario Pain Scale (CHEOPS), the Children’s and Infants Post Operative Pain Scale (CHIPPS), and the Objective Pain Scale (OPS) [20,23]. However, as Japanese hospitals do not currently use any of these observable scales, we thusly chose the VAS obs which is considered a simple assessment scale [24]. Our present results are in line with the original author’s results [3].

## Limitation

Our findings were limited by the use of a non-randomized participant pool that was chosen primarily by availability during the study period which may reduce the generalizability of our findings. Additionally, some numbers of measurements could not estimate patients pain, because of response to clinical emergency situation. We included various diagnostic categories to reflect intensive care settings but the resulting sample sizes might be insufficient for analyzing specific cohorts within each diagnostic condition.

## Conclusion

We established a novel Japanese version of the Face, Legs, Activity, Cry, Consolability (FLACC) Behavioral Scale through back-translation, and clinically tested for the patients in our PICU. High criterion validity and reliability were confirmed through our prospective study.

## Supporting information

S1 File This file contains all the data reported in the results. (XLSX)
